# Observations on Lithotomy

**Published:** 1811-10

**Authors:** Thomas Smith

**Affiliations:** Carlisle


*To the Editors of the Medical and Physical Journal.*

GENTLEMEN,

YOU did me the honour some time ago to insert some remarks of mine upon a paper by Mr. W. Simmons, a surgeon of Manchester, in your valuable Journal; and I then pledged myself to communicate, through the same useful medium, my experience of the use of the knife in Lithotomy, as recommended by those distinguished operators, Mr. Cooper, Mr. Blizzard, Mr. Gibson, and Mr. Lawrence; I am not, however, as yet prepared to offer the result of such a number of cases as I could wish, so many opportunities not having lately fallen to my share. You will, therefore, be pleased to consider my communication upon that subject as still in reserve, and in the mean time allow me to present a few observations upon a pamphlet of Mr. Simmons which only lately was made known to me. As it contains conclusions and suggestions tions upon the subject of lithotomy, which do not accord with my experience or that of many eminent writers, I hope you will favour me by communicating to the public my practical deductions upon Mr. S.'s cases and hints. In my former paper I took the liberty of addressing a few plain queries to Mr. S. which I thought that gentleman could have had no objection to have readily answered, for my information; since I had little doubt, from the general statement of his own success with the gorget, that his particular replies would have tended to support his general assertions upon that head. It appears, however, that Mr. S. either thinks it unnecessary or unpleasant to answer my inquiries; I must therefore look for similar information to another source; at the same time, I cannot help concluding both from Mr. S.'s silence and from the well-known failure of the operation in the hands of that gentleman, that his operations with the gorget have not been more exempt from accident and embarrassment than those of operators in general; and I am the more strongly confirmed in this conclusion, from the statement which Mr. S. himself has given of his total want of knowledge for many years of the situation of the membranous part of the urethra. So late as the year 1806, Mr. S. thought the acquirement of this knowledge a discovery worth communicating to the public, in a pamphlet entitled, "Hints for the more ready and safe Performance of the Operation for the Stone, &c." Such a title certainly raised hopes in me, which the contents of the pamphlet have failed to realize, for Mr. S. has divulged no new expedients to overcome the obstacles presented in those cases which he has related, and has failed to draw some conclusions from them, which appear to me both obvious and highly instructive. I trust therefore, by commenting upon them with candour and delicacy, my remarks will find a place in your valuable pages.

In his first case Mr. Simmons informs us, "that the membranous portion of the urethra was exposed by the *first* incision, and penetrated by the *second*, and that this part of the process did not occupy more than *half a minute*. But that the efforts employed in extracting the stone (which broke) and in using the scoop occupied about *half an hour*. The patient died about *fifty hours* after the operation." The bladder, when examined, was found "oblong in its shape, and reduced in its size to the thumb of a glove, and so thickened in its coats, as to excite rather the sensation of a solid piece of flesh, than a hollow viscus." The inner surface of the bladder, in short, exhibited strong appearances of irritation and inflammation. The hints which this case furnishes to to the profession are, in my opinion, very important; viz. that a surgeon should pay particular attention to divide, with the accuracy and deliberation of dissection, the external muscular parts concerned in lithotomy, instead of attempting to divide them by a single flourish of the knife. Mr. John Bell has, long ago, clearly explained how great an impediment this neglect of a proper division of the muscular parts forms to the extraction of a stone, and how apt a hard stone is to slip from the grasp of the forceps, or a soft stone to be crumbled to pieces by its blades. The extraction of a stone, however, after a careful division, will generally be performed without its breaking in the forceps, although it may be friable, nor will it be necessary to search in the bladder, with the scoop, for half an hour, in order to remove its fragments. The fatal symptoms which occurred in this case will likewise be avoided, as far as possible, as well as the risque of the after formation of a second stone, should the patient survive.

Mr. Simmons's second case contains no remark of greater importance than this, that in a case where no impediment was presented during the operation, symptoms of peritonæal inflammation may supervene, which an operator cannot account for, unless it be admitted that cold weather and a smoky town may have some influence in promoting these symptoms, even where " those material points of regard, the distension of the bladder and elevation of the nates, were attended to." I shall therefore pass on to the third case, in which Mr. S. informs us, that " the symptoms (of stone) were so violent as not to admit of delay, which otherwise would have been desirable, on account of the patient's recent escape from a severe attack of typhous fever, and of his still labouring under a white swelling of the knee joint, which had suppurated. Mr. Simmons's practice in this case appears singular, in as much, as it is a rule scarcely ever deviated from by judicious practitioners, " *not to perform the operation of lithotomy whilst the symptoms of what has been called a fit of the stone are urgent.*" Mr. Bromfield in his surgical remarks justly observes, " that if, during a fit of the stone in the bladder, the patient be cut, from the violent inflammation of the parts affected, it is not very improbable that the parts would sphacelate." Mr. S.'s case, therefore, practically furnishes us with a useful hint, that such a rule ought not to be deviated from, particularly under the previous and existing circumstances of the present case. For the probable consequence of this deviation was, that a few days after the operation " the patient's appetite began to fail him, he became hot and restless, his pulse quick and tongue foul, and the the wound, then on the point of healing, assumed an unfavourable aspect and shewed a disposition to spread." All these symptoms Mr. S. attributes, indeed, to the influence of smoke upon wounds, in confirmation of which he adds, "that no sooner had the coach, in which the patient was conveyed to country lodgings, passed without the (magic) circle of the smoke, which hung over the town, than the colour came into his lips." Whatever may be thought of the propriety of attributing these symptoms to such a cause as smoke, rather than to the debilitated and diseased state of the patient, and to the circumstance of operating during a fit of the stone, Mr. S. has certainly conveyed a very useful hint to all hospital surgeons, who were not fully aware of the fact before, viz. *that a private patient in the country is a much more eligible subject for lithotomy, than a patient in the hospital of a smoky town.*

Mr. Simmons's fourth case explains the inefficacy of attempting to turn out at once a stone from a contracted bladder, with the index of the left hand, and the impropriety of attempting to extract a stone so situated, by merely laying hold of its hither extremity, because (as in the present case) it may snap off. Why the contraction of the bladder, in this instance, should be spasmodic, because it appeared from the aspect of the stone extracted to have been of long standing, Mr. S. does not inform us.

I have thus taken the liberty of drawing a few conclusions from Mr. Simmons's cases, because I thought they would add considerably to the sum of information, which the relation of such cases convey to the public; and I shall now proceed to make some observations upon what Mr. Simmons calls his "Hints for the more ready and safe performance of the operation." Mr. S. first regrets that the descriptions given by practical writers of the true position of the membranous portion of the urethra are not very precise; "and perhaps less so, than the subject itself is susceptible off." To remedy this want of precision, Mr. S. after objecting to Mr. Earle's double staff, thus proceeds: "On taking a nearer view of the situation of these parts, it will be seen that in the erect position of the body, the membranous portion of the urethra lies under the center of the arch of the pubis, at a little distance from the symphysis, having the bulb situated at its anterior and the prostate gland situated at its posterior extremity. Making allowance, therefore, for any alteration of the parts from their natural position, occasioned either by the posture of the patient during the operation, or by the projection of the staff in perinæo, and aiming at the urethra on a line with the inner (quere *under)* surface of the symphysis symphysis pubis, that part of the membranous portion of the urethra, which lies contiguous to the prostate gland will be penetrated, and the incision may then be enlarged in the customary manner, so as to give an easy admission to the beak of the gorget into the groove of the staff. *Since this view of the subject presented itself to my mind, and which I first put in practice in the case of Devil and Pollit (in the year* 1806*) I have become possessed of a degree of confidence and comfort, while performing the operation of lithotomy, to which I was before a stranger. For the most part the membranous portion of the urethra has been nearly, if not entirely, exposed by the first incision, &c.*"

As Mr. Simmons promulgated this, because he thought he was thereby conveying a useful piece of information to the profession, it was certainly praise-worthy in him; and as the knowledge of the true position of the membranous part of the urethra was not conveyed to Mr. S. by any of the numerous surgical or anatomical writers who have pointed it out to a hair's breadth, nor had been ascertained by himself by dissection, Mr. S. would realize all the pleasure of a discoverer, in addition to the comfort and confidence, which, I confess, he would most naturally feel, from obtaining the knowledge of a point so absolutely indispensible to the performance of the operation of lithotomy, with any degree of safety. But, I apprehend, that it was totally useless to proclaim the circumstance, (as a hint for the more ready and safe performance of the operation,) which was so well known and had been so often explained. One proof of this out of many will suffice. It is selected from Mr. Earle's works, which Mr. S. had probably read, as he objects to Mr. Earle's double staff. "The bulb of the urethra," observes that accurate anatomist, "hangs pendulous before the arch of the pubes; the membranous part of the canal is a continuation of the urethra from the bulb to the entrance of the prostate gland; it is in length about three quarters of an inch, *and passes beneath the arch of the pubes*, unsurrounded by any thing, except cellular membrane. The membranous part of the urethra is not, however, in contact with the bone, but lies about three quarters of an inch beneath the arch, being connected to it by intervening cellular membrane." This description of the position of the membranous part of the urethra is somewhat more precise and clear than that given thirteen years afterwards by Mr. S., for it was published in the year 1793. It is, therefore, truly unfortunate, that Mr. Simmons did not chance to have read Mr. Earle's works, and made himself acquainted with a piece of anatomy so very essential to the "ready and safe performance of the operation," if, indeed, he performed the operation before the year 1806, which it is hardly charitable to suppose. If, however, he really operated in a few instances, it is easy to be conceived how much at random and how indecisive must have been the incisions of such an operator, and how entirely he must have depended upon the projection of the staff in perinæo; a guide which Mr. S. accordingly much extolls, although it is declared by the best operators to be a false guide, whose only tendency is effectually to derange the natural position of the parts in question. This derangement seems to have added a farther embarrassment to Mr. S.'s operations, and is erroneously attributed by him to the posture of the patient during operation. But, it may be asked, how can the posture of the patient derange the position of the membranous part of the urethra, or of the prostate gland? or how even derange the position of any of those muscles which arise from the pelvis itself, and are connected with parts which also are attached to the pelvis, although the patient, during Mr. S.'s operations, should writhe and twist his body ever so much?

This description of the position of the membranous part of the urethra conveys the only hint, which I can discover, in Mr. S's. pages, connected with the safe and ready performance of the operation; it was certainly a most essential piece of information, a most important discovery to an operator, the singularity of whose situation unfortunately consisted in the ignorance of it for many years. The knowledge of this fact, however, has lead Mr. Simmons to adopt and recommend a practice, as mischievous as any of those blunders which might be committed by an ignorance of it. For he ventures to inculcate the propriety of dividing (or rather attempting to divide) all the external parts at one incision! that he may penetrate the membranous part of the urethra, thus laid bare throughout the whole extent, or nearly so, by a second incision! Mr. S. may call this dexterous dispatch, if he pleases, but a well-judging, judicious operator, will rather calculate the time necessary to perform the operation by the comparatively deliberate steps necessary to that accurate dissection which the parts require, than by the movements of a stop watch. A steady operator will delight to rise from his seat rather with the substantial internal consciousness of having, by proper deliberation, insured the "safe and ready performance" of the operation, than with the transitory and mischievous gratification of having performed the most dangerous and most delicate of all operations, in a few minutes, with passing dexterity. I have been the more particular upon this point, because even Mr. S.'s example may tend to seduce some some young and thoughtless practitioner from the unostentatious paths of a steady operator, into an affected display of manual dexterity, so much to be deprecated in all surgical operations.

I have thus made a few observations upon Mr. Simmons's pages, which, I trust, will appear deficient neither in candour nor utility. For the instruction which I may have derived, he has my thankful acknowledgments, and I hope he will pardon the liberty I have taken, when he knows that my motives have been to render his communication more generally useful.

I remain, Gentlemen,

Your much obliged and obedient servant,

THOMAS SMITH.

*Carlisle, July 3d,* 1811.

